# Three-Dimensional-Printed GelMA-KerMA Composite Patches as an Innovative Platform for Potential Tissue Engineering of Tympanic Membrane Perforations

**DOI:** 10.3390/nano14070563

**Published:** 2024-03-23

**Authors:** Tuba Bedir, Dilruba Baykara, Ridvan Yildirim, Ayse Ceren Calikoglu Koyuncu, Ali Sahin, Elif Kaya, Gulgun Bosgelmez Tinaz, Mert Akin Insel, Murat Topuzogulları, Oguzhan Gunduz, Cem Bulent Ustundag, Roger Narayan

**Affiliations:** 1 Center for Nanotechnology and Biomaterials Application and Research (NBUAM), Marmara University, Istanbul 34722, Turkey; tubabedirr@gmail.com (T.B.); baykaradilruba@gmail.com (D.B.); aceren@marmara.edu.tr (A.C.C.K.); oguzhan@marmara.edu.tr (O.G.); 2 Department of Metallurgical and Materials Engineering, Faculty of Technology, Marmara University, Istanbul 34722, Turkey; 3 Department of Biochemistry, Faculty of Medicine, Marmara University, Istanbul 34722, Turkey; alisahin@marmara.edu.tr; 4 Department of Basic Pharmaceutical Sciences, Faculty of Pharmacy, Marmara University, Istanbul 34668, Turkey; marunelif@gmail.com (E.K.); gulgun.tinaz@marmara.edu.tr (G.B.T.); 5 Department of Chemical Engineering, Faculty of Chemical and Metallurgical Engineering, Yildiz Technical University, Istanbul 34210, Turkey; makinsel@yildiz.edu.tr; 6 Department of Bioengineering, Faculty of Chemical and Metallurgical Engineering, Yildiz Technical University, Istanbul 34210, Turkey; mtopuz@yildiz.edu.tr; 7 Health Biotechnology Joint Research and Application Center of Excellence, Istanbul 34220, Turkey; 8 Joint Department of Biomedical Engineering, University of North Carolina, Chapel Hill, NC 27599, USA

**Keywords:** tympanic membrane perforation, gelatin methacryloyl, keratin methacrylolyl, DLP 3D printing, nanoparticle, gentamicin, FGF-2

## Abstract

Tympanic membrane (TM) perforations, primarily induced by middle ear infections, the introduction of foreign objects into the ear, and acoustic trauma, lead to hearing abnormalities and ear infections. We describe the design and fabrication of a novel composite patch containing photocrosslinkable gelatin methacryloyl (GelMA) and keratin methacryloyl (KerMA) hydrogels. GelMA-KerMA patches containing conical microneedles in their design were developed using the digital light processing (DLP) 3D printing approach. Following this, the patches were biofunctionalized by applying a coaxial coating with PVA nanoparticles loaded with gentamicin (GEN) and fibroblast growth factor (FGF-2) with the Electrohydrodynamic Atomization (EHDA) method. The developed nanoparticle-coated 3D-printed patches were evaluated in terms of their chemical, morphological, mechanical, swelling, and degradation behavior. In addition, the GEN and FGF-2 release profiles, antimicrobial properties, and biocompatibility of the patches were examined in vitro. The morphological assessment verified the successful fabrication and nanoparticle coating of the 3D-printed GelMA-KerMA patches. The outcomes of antibacterial tests demonstrated that GEN@PVA/GelMA-KerMA patches exhibited substantial antibacterial efficacy against *Staphylococcus aureus*, *Pseudomonas aeruginosa*, and *Escherichia coli*. Furthermore, cell culture studies revealed that GelMA-KerMA patches were biocompatible with human adipose-derived mesenchymal stem cells (hADMSC) and supported cell attachment and proliferation without any cytotoxicity. These findings indicated that biofunctional 3D-printed GelMA-KerMA patches have the potential to be a promising therapeutic approach for addressing TM perforations.

## 1. Introduction

The tympanic membrane (TM), also referred to as the eardrum, is a tissue that is concave, thin (~0.1 mm thick), and positioned at the end of the ear canal, which separates the middle ear compartment from the outer ear [1]. The essential role of the TM is to convey sound waves as vibrations to the inner ear through ossicular chains, as well as to protect the middle ear [2]. The leading causes of TM perforations are middle ear infections, acoustic trauma, and foreign body insertion into the ear, resulting in hearing loss and infection in the human ear due to the loss of the vibrating surface and the loss of the protective barrier’s functionality, respectively [1,3]. Most acute perforations have the potential to heal spontaneously without an external stimulus, and can heal within a week to a month [4]. When perforations do not heal naturally and persist for more than three months, they are classified as chronic. Surgical intervention (myringoplasty or tympanoplasty) is necessary to repair chronic perforations [2,5]. Typically, absorbable scaffold materials like rice paper, fat, or Gelfoam are used in performing a myringoplasty, which is a surgical procedure for small central perforations [6]. In the case of an unsuccessful myringoplasty or large chronic perforations, tympanoplasty using autologous grafts (fascia or perichondrium) is usually carried out in an operating room [7]. Despite surgical procedures being the best treatment for the current condition, they have drawbacks, such as the necessity for general anesthesia, the requirement for the surgeon to have special microsurgical skills, and the high cost of the surgery [6,8]. In addition, re-perforations or failures may necessitate multiple surgeries, leading to donor site morbidity and limited access to autologous graft materials [6,9]. To address the shortcomings of surgical treatment and accelerate the recovery process for chronic TM perforations, it is necessary to develop novel therapies that utilize TM scaffolds that exhibit functional material properties and 3D geometries.

Tissue engineering is an emerging interdisciplinary area that has the potential to overcome the drawbacks and constraints associated with existing surgical methods in TM perforations. It encompasses the use of cells, biomaterial scaffolds, signaling molecules, and growth factors to reinstate the structure and functionality of the TM [4,10]. Three-dimensional printing technology is considered an attractive method for producing 3D tissues and organs, making it a promising technique for tissue engineering applications [11]. It provides key advantages, such as precisely controllable geometry and porosity, the fabrication of complex structures, low cost, and high reproducibility [12,13]. Extrusion-based and laser-based techniques are extensively utilized among the various 3D printing methods; however, extrusion-based printing has drawbacks such as restricted printing speeds, imperfect structure, and limited printing resolution which are associated with the small nozzle diameter [14,15]. To overcome these challenges, digital light processing (DLP)-based 3D printing employs ultraviolet (UV) light through a projector to cure the photopolymer layer by layer in accordance with computer-aided design (CAD). Using a projector allows each full layer to be cured in one go, resulting in faster print times [16]. Thus, DLP allows complex structures to be produced with a higher printing speed, resolution, and cell viability than other 3D printing approaches [17].

Criteria such as printability, biocompatibility, and mechanical properties should be considered when selecting the appropriate biomaterial ink for DLP printing [18]. At present, the large number of inks utilized in light-based 3D bioprinting includes photoreactive biomaterials that undergo crosslinking and transform into hydrogels through free-radical polymerization, which is triggered by light initiation [17]. Photoreactive biomaterials including silk fibroin methacrylate (SilMA) [18], hyaluronic acid methacrylate (HAMA) [19], and poly (ethylene glycol) diacrylate (PEGDA) [20] are extensively employed; gelatin methacryloyl (GelMA) is the most prevalent ink choice used for light-based 3D bioprinting [21]. GelMA formed by the grafting of methacrylic anhydride onto the amine-containing side groups of gelatin exhibits excellent photocurability and can be crosslinked by exposure to UV in the presence of photoinitiators [22]. This material exhibits an outstanding biocompatibility, good biodegradability, low antigenicity, and tunable physical properties [23]. Also, the potential of incorporating cell-supporting moieties such as Arg-Gly-Asp (RGD) makes GelMA an ideal platform for cell growth [24]. Nevertheless, the limited mechanical properties of GelMA hydrogel significantly restrict its potential applications in microscale construction [25]. The 3D scaffold of GelMA with enhanced mechanical properties and extra properties for tissue engineering (e.g., better formability, biocompatibility) can be formed by crosslinking the functional groups of GelMA with other polymers, small organic molecules, and inorganic particles [26].

Keratin is a structural protein with a fibrous morphology that is found in hair, nails, feathers, horns, hooves, wool, and epithelial coverings [27]. It exhibits a high amount of sulfur-containing groups because of the presence of cysteine-rich proteins, which form a characteristic fibrous structure through disulfide bonds; this material is associated with good mechanical properties and stability [28]. It also comprises cell-binding motifs, including glutamic acid-aspartic acid-serine (EDS), leucine-aspartic acid-valine (LVD), and arginine-glycine-aspartic acid (RGD), which encourage cell attachment and cell proliferation [29]. Due to its good biocompatibility and biodegradability, there is increasing interest in the use of keratin hydrogels in tissue engineering scaffolds [30,31]. Scaffolds with high mechanical properties and biocompatibility for TM perforations can be obtained by crosslinking GelMA and methacrylated keratin (KerMA).

Growth factors are naturally existing biomolecules that play a role in stimulating cellular growth and accelerating wound healing. Recent studies have indicated that growth factors possess the ability to promote the recovery of the perforated TM. FGF-2 (fibroblast growth factor-2) is one of the most commonly used growth factors in the repair of TM perforation [2,4]. The closure of traumatic TM perforation using FGF-2 has demonstrated a notable success rate among patients [32]. Moreover, various studies in humans have indicated that combining FGF-2 with different scaffold materials results in a more rapid and improved recovery rate [33,34]. Hence, incorporating growth factors into the scaffold can enhance the ability of TM perforations to heal.

Polymeric micro/nanoparticles have been extensively investigated as biodegradable vehicles for the controlled administration and sustained release of several types of therapeutic agents, such as drugs, genes, and proteins [35]. Specifically, poly (vinyl alcohol) (PVA) is an FDA-approved, water-soluble, and non-toxic biodegradable polymer; several drugs have been encapsulated in PVA particles [36]. The use of antibiotic nanoparticle compositions provides several benefits, including targeted drug delivery, simplified dosage regimes for prolonged antibiotic release, and masking of the encapsulated drug by reducing the risk of systemic toxicity [37,38]. Gentamicin (GEN), an aminoglycoside antibiotic agent that is recognized for its wide range of antibacterial activities, prevents the synthesis of bacterial proteins by attaching to the 30S subunit sections of the bacterial ribosome [39]. It is the favored antibiotic for addressing bacterial infections that are associated with microorganisms, including *Pseudomonas aeruginosa*, *Escherichia coli*, and *Staphylococcus aureus* [40]. Utilizing nanoparticles to deliver GEN can be an efficient strategy for a drug delivery system which can reduce the side effects of the drug and potentially extend its activity [41].

Electrohydrodynamic atomization (EHDA), also known as the electrospray process, has emerged as a promising technology for preparing polymeric micro/nanoparticles for drug delivery [42]. In an electrospray process, an electric field that is applied to a charged liquid droplet exiting the capillary can serve to deform the interface, which generates a Taylor cone. The electrostatic force produced by the application of a high voltage helps to overcome the surface tension of the droplet by sputtering the particles. Eventually, the solvent evaporates and the solid micro/nanoparticles can be collected [43]. The desired particle shape and particle size can be obtained by regulating the flow velocity, voltage, solution concentration, and electrical conductivity [44]. Moreover, this method offers benefits such as one-step processing, high reproducibility, and high efficiency [45]. In addition, coaxial electrospray, an advanced technology to create core-shell particles, sprays outer and inner solutions simultaneously from two separate feed channels into a coaxial nozzle [46].

**Figure 1:**
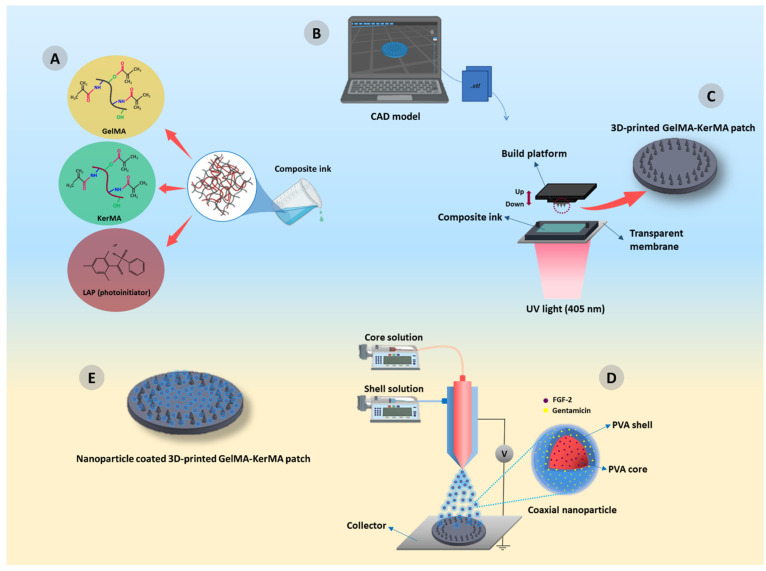
Schematic diagram of nanoparticle coated-GelMA-KerMA patches development process: (**A**) Preparation of GelMA-KerMA composite ink; (**B**) CAD file preparation and slicing for the 3D printing process; (**C**) 3D printing of GelMA-KerMA patches containing microneedles; (**D**) the process of coating GelMA-KerMA patches with coaxial nanoparticles by EHDA method; (**E**) final version of the coaxial nanoparticle coated 3D-printed GelMA-KerMA patch.

Here, we report the design and production of an innovative composite patch using photocrosslinkable GelMA and KerMA hydrogels to present a new tissue engineering approach for TM perforations. The edges of the GelMA-KerMA patches, designed in a circle, are equipped with conical microneedles for better attachment to the perforation area. First, 3D-printed GelMA-KerMA patches equipped with microneedles were obtained using the DLP 3D printing method. Later, the patches gained biofunctionality by coaxially coating with GEN and FGF-2-loaded PVA nanoparticles using the EHDA method (Figure 1). As far as we know, this study is the first instance in the literature to use the combination of both GelMA and KerMA. In addition, coating the surface of GelMA-KerMA patches with coaxial nanoparticles is another novelty of this study. The physicochemical and mechanical properties of the nanoparticle-coated GelMA-KerMA patches were characterized, including their morphology, a mechanical analysis, and their swelling and degradation capabilities, as well as their drug release profiles. After that, the antimicrobial properties and biocompatibility of the patches were evaluated in vitro. These custom-designed innovative 3D-printed patches can present a hopeful strategy for tissue engineering of TM perforations.

## 2. Materials and Methods

### 2.1. Materials

Gelatin (Type A, gel strength ~300 g Bloom), methacrylic anhydride (MAA), lithium phenyl-2,4,6-trimethyl-benzoyl phosphinate (LAP), polyvinyl alcohol (PVA, *M_w_* = 130.000 g/mol), gentamicin sulfate, FGF-2 human, glutaraldehyde solution (GA, 50 wt%. *M_w_* = 100.12 g/mol), and dialysis membrane (average flat width 43 mm, cut-off value 14 kDa) were acquired from Sigma–Aldrich (Sigma–Aldrich, Darmstadt, Germany). An FGF-2 ELISA kit was purchased from BT LAB (BT LAB, Jiaxing, China). Sodium hydroxide, sodium carbonate, and fuming hydrochloric acid 37% were acquired from Merck KGaA (Merck KGaA, Darmstadt, Germany). Sodium hydrogen carbonate (>99.7%) was acquired from ISOLAB (ISOLAB, Eschau, Germany). Phosphate-buffered saline (PBS, pH 7.4) and tris buffer (pH 7.6) were obtained from ChemBio (ChemBio, Istanbul, Turkey). Sheep wool was obtained from Kazlıcesme R&D Centre and Test Laboratory (Kazlıcesme R&D Centre and Test Laboratory, Istanbul, Turkey). Urea (extrapure) was purchased from Tekkim Kimya (Tekkim Kimya, Bursa, Turkey). l-cysteine was obtained from Biosynth (Biosynth Ltd., Compton, UK).

### 2.2. Synthesis and Characterization of Gelatin Methacryloyl (GelMA)

The GelMA synthesis method has been discussed in detail previously [24]. In short, 10% (*w*/*v*) gelatin solution of type A porcine skin was dissolved in 0.1 M carbonate bicarbonate buffer system (5.86 g sodium bicarbonate and 3.18 g sodium carbonate in 1 L of distilled water, pH 9) at 60 °C under vigorous stirring. Then, 0.1 mL of methacrylic anhydride (MAA) per gram of gelatin was split into six equal volumes and added in a dropwise manner to the solution under vigorous stirring every 30 min for 3 h at 50 °C and pH was readjusted to a value of 9–9.5 following each addition. Next, the reaction was terminated via adjustment of the pH value to 7.4. Following this, the solution was dialyzed against distilled water at 40 °C for 5 days using a 14 kDa molecular-weight-cutoff (MWCO) membrane in order to remove unreacted MAA and methacrylic acid byproducts. Finally, the resulting solution was lyophilized in a freeze-dryer for 3 days and stored at 4 °C for further use.

^1^H NMR spectroscopy (Bruker Avance III 600 MHz, Bremen, Germany) was conducted to investigate the functionalizing gelatin with methacryloyl groups. Gelatin and GelMA samples were separately dissolved in D_2_O at 10 mg/L. ^1^H NMR spectra were acquired at room temperature at a frequency of 600 MHz.

### 2.3. Extraction of Keratin from Wool

The process of extracting keratin from sheep wool was conducted with modifications based on the studies of Wang et al. [47] and He et al. [48]. Firstly, any large impurities in the wool were removed manually; next, it was washed with detergent and rinsed with deionized water. After overnight drying, 100 g of the rinsed wool was immersed in a 1:1 mixture of acetone/ethanol (1 L) for 24 h. Subsequently, it was thoroughly rinsed with deionized water and then dried in an oven at 60 °C for one night. Prior to extraction, the pieces of wool were cut into several millimeters in length, and 10 g of wool was weighed and slowly added to a solution prepared with 8 M urea and 0.165 M l-cysteine in 5 M NaOH at pH 10.5, previously heated to 60 C. After the last piece of wool was added, the temperature was raised to 75 °C, and the extraction was allowed to proceed under stirring for at least 5 h. After the reaction, the mixture was first filtered through a muslin cloth using vacuum filtration. Then, it was passed through a general-purpose filter paper and finally through a fast qualitative filter paper (MN 617, Macherey-Nagel, Allentown, PA, USA). The filtered extract was transferred to a dialysis membrane and dialyzed against deionized water, changing the water four times a day for a minimum of three days. After dialysis, the keratin solution was frozen at −20 °C for 24 h and subsequently at −80 °C for another 24 h before being lyophilized (−58 °C, <0.1 mbar) to obtain keratin powder.

### 2.4. Synthesis and Characterization of Keratin Methacryloyl (KerMA)

Methacrylic anhydride was used for the modification of keratin by introducing methacryloyl functional groups. First, keratin 5% (*w*/*v*) was dissolved in a slightly warmed 0.25 M bicarbonate buffer (pH 9.0 at 50 °C). After reaching a temperature of 50 °C, methacrylic anhydride was added in a dropwise manner to the solution; the reaction was allowed to continue for 3 h. The pH value of the solution was monitored at the beginning, middle, and end of the reaction. After adjusting the pH of the solution to 7.0 to stop the reaction, the resulting solution was dialyzed against deionized water, changing the water four times a day for at least 5 days using dialysis membranes (with MWCO 12–14 kDa). Following dialysis, the functionalized keratin solution was frozen at −20 °C for one day and at −80 °C for another day, then lyophilized (at −58 °C, <0.1 mbar) for 3 days to obtain a dry powder. The lyophilized keratin with methacrylate was stored at −20 °C until use.

The functionalization of keratin with methacryloyl groups was confirmed using ^1^H NMR spectroscopy (Bruker Avance III 600 MHz, Bremen, Germany). Keratin and KerMA samples were separately dissolved in D_2_O at 10 mg/mL. ^1^H NMR spectra were acquired at a frequency of 600 MHz at room temperature.

### 2.5. Preparation of GelMA-KerMA Composite Ink

To prepare GelMA-KerMA composite ink, GelMA and KerMA solutions with mass fractions of 32/8% (*w*/*v*) were prepared by dissolving in PBS (pH 7.4) under constant stirring at 40 °C. The photoinitiator LAP was added to the composite ink at a concentration of 0.25% (*w*/*v*) for 10 min. Later, the obtained ink was cooled to room temperature and subsequently transferred to the vat of the DLP printer for 3D printing.

### 2.6. Design and Fabrication of 3D-Printed GelMA-KerMA Patches

The patches of GelMA-KerMA composite ink were processed using a 3D printer (Phrozen Sonic Mini 8K, Phrozen Tech Co., Ltd., Hsinchu, Taiwan). First, the patch model was constructed using computer-aided design (CAD) software in the form of the SolidWorks 2020 SP05 (Dassault Systèmes SE, Vélizy-Villacoublay, France) program. GelMA-KerMA composite patches were designed with a diameter of 10 mm and a height of 100 µm; the edges of the patches contained conical microneedles with a height of 200 µm. Next, CAD files of the patches were converted to the .stl file format and then sliced using the software of the 3D printer, Chitubox (Shenzhen Chuangbide Technology Co., Ltd., Shenzhen, China). The slices were solidified using the 3D printer in order to create the designed patches. Patches were printed with a 20 μm layer thickness and 90 s exposure time by controlling the following other printing parameters: light intensity of 12 mW/cm^2^ and light wavelength of 405 nm. After printing, 3D printed patches were dried in the dark for 24 h at room temperature and stored in a dehumidified container for further use.

### 2.7. Nanoparticle Coating of GelMA-KerMA Patches by Coaxial Electrospray Method

In this study, core–shell nanoparticles were produced by the coaxial electrospray method. A laboratory-scale electrospraying device (Inovenso, Istanbul, Turkey) was used to perform coaxial electrospraying. The electrospray system consists of two syringe pumps, a stainless-steel nozzle containing a coaxial needle, a high-voltage DC power supply, and a collector. The coaxial needle included a 22-gauge inner needle and an 18-gauge outer needle.

GEN-loaded PVA was prepared as the shell solution, and FGF-2-loaded PVA was prepared as the core solution for the coaxial electrospraying process. Briefly, 1% (*w*/*v*) PVA was prepared in distilled water/ethanol mixture with a ratio of 9:1 (*v*/*v*) for 1 h at 95 °C and 0.2% (*w*/*v*) GEN was added to the PVA solution under constant stirring. To prepare the core solution, 100 ng/mL of FGF-2 was added to the 1% (*w*/*v*) PVA solution and vortexed. Next, FGF-2@PVA (core) and GEN@PVA (shell) solutions were fed into two 10 mL plastic syringes that were attached to the coaxial needle. Two pumps were applied to deliver the GEN@PVA and FGF-2@PVA solutions through a coaxial system. FGF-2@PVA solution was sprayed at a flow rate of 0.1–0.2 mL/h and GEN@PVA at a flow rate of 0.2–0.3 mL/h. The applied voltage was set to 21–25 kV; the distance between the collector and the needle tip was 15 cm. Later, 3D-printed GelMA-KerMA patches were placed on the collector and coated with GEN@PVA and FGF-2@PVA nanoparticles as single axial and coaxial for 3 h. All electrospraying processes were carried out under ambient conditions. Finally, the nanoparticle-coated patches were placed in a desiccator with glutaraldehyde solution and crosslinked with glutaraldehyde vapor in an oven at 40 °C for 1 h before characterizations were performed. Details of the electrosprayed samples are listed in Table 1.

### 2.8. Fourier Transform Infrared Spectroscopy (FTIR)

A Fourier-transform infrared spectroscopy instrument (FTIR, FT/IR-ATR 4700, Jasco, Easton, MD, USA) was utilized to analyze the chemical structure of GelMA, KerMA, blank GelMA-KerMA, and nanoparticle-coated GelMA-KerMA patches. FTIR spectra were obtained in the scanning range of 4000–400 cm^−1^; the spectra were acquired with a resolution of 4 cm^−1^.

### 2.9. Morphological Properties of GelMA-KerMA Patches

The shape, dimensions, and morphology of blank GelMA-KerMA and nanoparticle-coated GelMA-KerMA patches were examined with a scanning electron microscope (SEM) (EVA MA 10, Zeiss, Jena, Germany). Before imaging, gold coating was performed on all patches with a sputter coating machine (Quorum SC7620, ABD). The average particle diameter of the nanoparticles on the patches was calculated with Image J 1.53v software (National Institutes of Health, Bethesda, MD, USA). At least 100 particles from SEM images were used to measure diameters.

### 2.10. Mechanical Characteristics of GelMA-KerMA Hydrogels

The mechanical properties of the fresh hydrogels of blank GelMA-KerMA and nanoparticle-coated GelMA-KerMA were assessed using a compression testing machine equipped with a 5 kN load cell (EZ-LX, Shimadzu, Kyoto, Japan). For compression analyses, cylindrical hydrogels with dimensions of 6 mm in height and 8 mm in diameter were fabricated. Compression tests were executed to a maximum strain of 60% at a rate of 1 mm/min. The compressive modulus was determined as the slope of the linear region (0–20%) of the stress–strain curve.

### 2.11. Swelling and Degradation Analysis for GelMA-KerMA Patches

To characterize the swelling and degradation properties of blank GelMA-KerMA and nanoparticle-coated GelMA-KerMA patches, the patches were studied in PBS (pH 7.4) at 37 °C at certain time intervals. For the swelling analysis, the dried samples were immersed in PBS for 5.5 h at specific time intervals (5, 10, 20, 40, 60, 90, 150, 210, and 330 min). The swelling ratio of the patches was calculated with the following Equation (1):

(1) $$S=\frac{Ww-W_{d}}{W_{d}}\times 100$$

In this equation, S is the swelling ratio, *W_d_* is the weight of the patches before immersion in PBS, and *W_w_* is the weight of the patches after water uptake for specific time intervals. All experiments were performed in triplicate.

It is crucial to model the swelling kinetics of hydrogels in order to comprehend the nature and rate of water diffusion into the hydrogel as well as the forces that influence the swelling of the hydrogel. Fick’s laws are the most frequently utilized relationships for representing the swelling kinetics of hydrogels. Equation (2) is an application of Fick’s law specifically for reticulated, swellable polymers [49,50].

(2) $$\frac{W_{w}}{W_{e}}=kt^{n}$$

In this equation, $W_{e}$ is the weight of hydrogels at equilibrium, *k* is a specific rate constant, and *n* is the exponent characteristic associated with the mode of transport of the solute. For a value of *n* = 0.5, the solute diffuses through the polymer and is released using a quasi-Fickian diffusion mechanism, even if the polymer undergoes dynamic swelling and the associated macromolecular relaxations. Non-Fickian solute diffusion is observed at values of *n* > 0.5. The unique scenario where *n* = 1 has garnered interest due to the possible applications of this solute transport phenomenon in zeroth-order release and swelling-controlled systems of bioactive substances. This method of solute transportation can be referred to as pseudo-case-II solute transportation. To model the swelling behavior of the hydrogels, the extension of the above Fick’s law (see Equation (3) [49,51] is integrated to yield the simplified Equation (4).

(3) $$\frac{dS\left(t\right)}{dt}=k_{s}^{\prime }\;{\left(S_{e}-S\left(t\right)\right)}^{n}$$

(4) $$S\left(t\right)=S_{e}-k_{s}\;t^{-m}$$

In this equation, $S_{e}$ is swelling at equilibrium, $k_{s}^{\prime }$ and $k_{s}$ are rate constants, and n and m are exponents. In this study, MATLAB R2023a is utilized to obtain the model coefficients $S_{e}$, $k_{s}$, and m, which are also meaningful as they contain direct information about the kinetics. $S_{e}$ is related to the maximum swelling of the hydrogels, while $k_{s}$ and m directly inform us about the kinetics. Lower $k_{s}$ and higher m values indicate a faster attainment of equilibrium.

To validate the obtained models, correlation coefficient (*R*^2^) and root mean square error (RMSE) values are utilized, as given in Equations (5) and (6) [52,53]. The *R*^2^ value shows how well the model is correlated with the data, and the root mean square error (RMSE) value quantifies the average discrepancy between the predictions generated by a model and the actual values, providing an assessment of the ability of the model to accurately estimate the target value.

(5) $$R^{2}=1-\left[\frac{\sum_{i=1}^{m}{\left(y_{i}-{\hat{y}}_{i}\right)}^{2}}{\sum_{i=1}^{m}{\left(y_{i}-{\bar{y}}_{i}\right)}^{2}}\right]$$

(6) $$RMSE=\sqrt{\frac{\sum_{i=1}^{m}{\left(y_{i}-{\hat{y}}_{i}\right)}^{2}}{m}}$$

The degradation performance of the patches was determined for 5 days by weighing the samples at specific time points (3, 6, 9, 15, 24, 48, 72, and 120 h). The samples were immersed in PBS, taken out at a certain time, dried, and weighed. The degradation rate of the patches was calculated according to Equation (7):

(7) $$DR=\frac{W_{d}-W_{t}}{W_{d}}\times 100$$

In this equation, DR is the degradation rate, *W_d_* is the weight of the patches before immersion in PBS, and *W_t_* is the dry weight of the patches after a specific incubation time. All measurements were performed in triplicate.

### 2.12. In Vitro GEN Release Study

In vitro release profiles of GEN from GEN@PVA/GelMA-KerMA patches were performed in PBS (pH 7.4). The linear calibration curve of GEN at different concentrations (0.2, 0.4, 0.6, 0.8, and 1 µg/mL) was determined over the wavelength range of 190–400 nm. GEN@PVA/GelMA-KerMA patches were incubated in 1 mL of PBS (pH 7.4) in a thermal shaker (BIOSAN TS-100, Riga, Latvia) at 37 °C. UV–Vis spectroscopy (Jenway 7315, Bibby Scientific, Staffordshire, UK) was utilized to detect the released amounts of GEN by measuring the absorbance value at 195 nm. After each measurement at predetermined time points (0.25, 0.5, 1, 2, 3, 4, 6, 8, 12, 24, 48, 72, and 96 h), 1 mL of PBS was swapped for a fresh one.

### 2.13. In Vitro FGF-2 Release Study

To evaluate the in vitro release profile of FGF-2, FGF-2@PVA/GelMA-KerMA patches were incubated in PBS (pH 7.4) on an orbital shaker at 37 °C. At predetermined time points (15, 30 min; 1, 3, 6, 12 h; 1, 2, and 4 days), 500 µL of the release solution from each sample was collected; 500 µL of fresh PBS was then added. The collected solutions were stored at −20 °C until they were used. The FGF-2 levels in the collected solutions were quantified by the FGF-2 ELISA kit according to the manufacturer’s instructions.

### 2.14. GEN Release Kinetics

The mechanism of drug release kinetics from the GEN@PVA/GelMA-KerMA patches was investigated using the five most commonly utilized mathematical kinetic models: the Korsmeyer–Peppas model, the zero-order model, the first-order model, the Higuchi model, and the Hixson–Crowell model. The equations of the Korsmeyer–Peppas model, zero-order model, first-order model, Higuchi model, and Hixson–Crowell model were presented as follows, respectively.

*Q* = *Kt^n^* (8)

*Q* = *K*_0_*t* (9)

*In* (1 − *Q*) = −*K*_1_*t* (10)

*Q* = *K_h_t*^1/2^ (11)

*Q*^1/3^ = *K_hc_t* (12)

In these mathematical equations, the kinetic constants are *K*_0_, *K*_1_, *K*, *K_h_*, and *K_hc_*; *Q* is the fractional amount of drug release at time *t*. In addition, *n* is the diffusion exponent that is indicative of the drug release mechanism.

### 2.15. In Vitro Antimicrobial Activity

The antibacterial efficacy of GelMA-KerMA and GEN@PVA/GelMA-KerMA patches was assessed against *S. aureus* ATCC 25923 and *P. aeruginosa* 27853 and *E. coli* ATCC 25922 strains using the disc diffusion method recommended by the Clinical Laboratory Standards Institute (CLSI). Overnight cultures of *S. aureus* ATCC 25923, *P. aeruginosa* 27853, and *E. coli* ATCC 25922 were spread onto Mueller–Hinton Agar plates. Subsequently, the prepared 8 mm discs were positioned on the agar plates; the discs were incubated at 37 °C for 24 h. The inhibition zones surrounding the discs were then measured using a ruler to determine the extent of antibacterial activity.

### 2.16. Cell Culture Studies

#### 2.16.1. Cell Viability

The growth of human adipose-derived mesenchymal stem cells (hADMSC) was tested on the produced GelMA-KerMA patches for 1, 3, and 7 days. GelMA-KerMA, GEN@PVA/GelMA-KerMA, FGF-2@PVA/GelMA-KerMA, and FGF-2@GEN@PVA/GelMA-KerMA patches were placed into a 96 well-plate and then subjected to 20 min of UV radiation for sterilization. The cells (hADMSCs) were seeded on 96 well plates at a seeding density of 1 × 10^4^ cells/mL in DMEM containing 10% fetal bovine serum and 0.1 mg/mL penicillin/streptomycin; the cells were then placed in a humidified incubator at 37 °C with 5% CO_2_. To establish a baseline for assessing cell culture functionality, a standard tissue culture polystyrene (TCPS) plate was employed as the control. The MTT (3-(4, 5-dimethylthiazolyl-2)-2, 5-diphenyltetrazolium bromide) cell proliferation assay was utilized to investigate cytotoxicity at a given time point. The test was performed according to the protocol that was provided by the manufacturer; the absorbance values were measured at 570 nm wavelength (690 nm as reference value) using an ELISA plate reader (Enspire, Perkin Elmer, Waltham, MA, USA). The samples for the assay were studied in triplicate.

#### 2.16.2. Fluorescence Microscopy Analyses

To evaluate the attachment of hDMSCs on GelMA-KerMA patches, DAPI staining was undertaken on day 7 of culture. First, the growth medium was discarded, and patches were rinsed three times with 100 μL of pre-warmed PBS. After that, the cells were fixed with 4% formaldehyde (Sigma–Aldrich, Darmstadt, Germany) at room temperature (RT) for 30 min, rinsed with PBS, and permeabilized in 0.1% Triton X-100 (Merck KGaA, Darmstadt, Germany) at RT for 10 min. Next, the samples were incubated in 1 μg/mL DAPI (Invitrogen, Waltham, MA, USA) for 20 min at RT to stain the nuclei of the cells. Finally, the DAPI solution was removed, and patches were observed under an inverted fluorescence microscope (Leica Microsystems Inc., Deerfield, IL, USA).

#### 2.16.3. SEM Characterization

Morphological analyses of hADMSCs on patches were characterized using SEM. After 7 days of incubation, the samples were fixed using 2.5% glutaraldehyde (Sigma, St. Louis, MO, USA) for 1 h; the samples were subsequently dehydrated via serial dilutions of ethanol (30%, 50%, 70%, 90%, and 99%). Dried samples were sputter-coated with gold for 90 s and analyzed by SEM using an accelerating voltage of 10 kV.

### 2.17. Statistical Analysis

The experiments were conducted at least in triplicate; the data are expressed as mean ± standard deviation (SD). Post hoc one-way ANOVA using a Tukey–Kramer pair-wise comparison was utilized for statistical analysis. A value of *p* ≤ 0.05 is considered to be statistically significant; additional significance is indicated by ** for *p* < 0.01 and *** for *p* < 0.001.

## 3. Results and Discussion

### 3.1. H NMR Analysis of GelMA and KerMA

**Figure 2:**
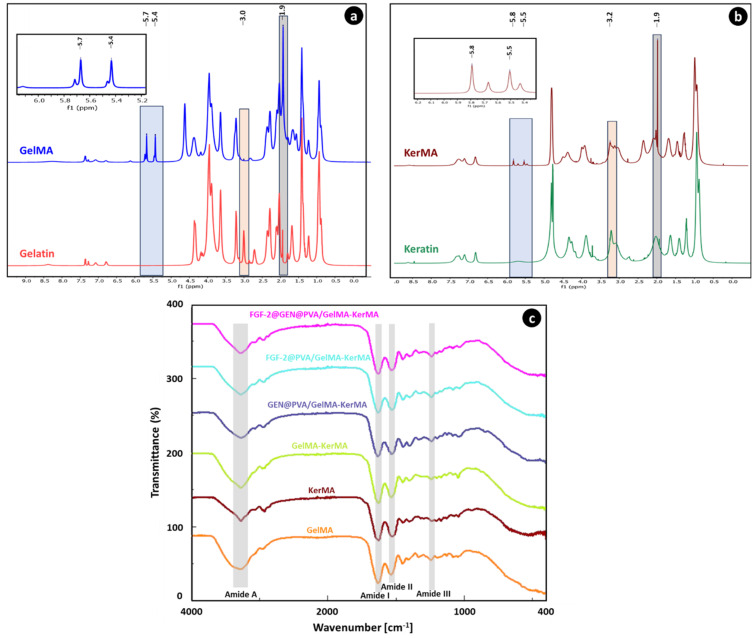
(**a**) ^1^H-NMR spectra of gelatin and GelMA. Peaks observed at 5.7 ppm, 5.4 ppm, and 1.9 ppm indicate the presence of methacrylation in the protein. The peak at 3.0 ppm signifies a reduction in lysine groups. (**b**) ^1^H-NMR spectra of keratin and KerMA. Peaks observed at 5.8 ppm, 5.5 ppm, and 1.9 ppm indicate the presence of methacrylation in the protein. The peak between 3.0 and 3.3 ppm may signify a reduction in lysine groups. (**c**) FTIR spectra of GelMA-KerMA patches.

The successful functionalization of amine groups in the gelatin structure with methacrylate groups was confirmed by ^1^H-NMR analysis. According to Figure 2a, when compared to the spectrum of gelatin, the GelMA revealed additional signals that can be attributed to the presence of methacryloyl groups. New signals from about 5.4 to 5.7 ppm in the GelMA spectrum are assigned to acrylic protons (2H) of the methacryloyl group grafted to the lysine and hydroxylysine residues associated with the gelatin backbone. This proved the presence of a C=C bond associated with vinyl groups of methacrylate anhydride in the structure of the anhydride [54]. Further confirming this finding, the GelMA spectrum shows a reduction in the peak corresponding to the lysine methylene (2H) at around 3.0 ppm [55]. Additionally, the peak intensity in the range of 1.9 ppm is related to methyl protons (3H) of the methacryloyl groups [56].

^1^H-NMR analysis was conducted to confirm the modification of keratin protein with methacrylate agents (Figure 2b). Upon analyzing the ^1^H-NMR spectra of keratin and KerMA, it was found that the peaks at around 5.5 and 5.8 ppm correspond to the acrylic protons of the methacrylamide groups that were formed during methacrylation, with the presence of methyl protons at approximately 1.9 ppm. The peak between 3.0 and 3.3 ppm may signify a reduction in the number of lysine groups. Some studies have reported the presence of an additional peak at 6.1 ppm, which suggests that methacrylation may occur through the hydroxyl groups, possibly due to the excessive use of methacrylation agents [24,57,58]. However, in our study, the absence of any peak at this value suggests that methacrylation predominantly occurs via amine groups. In a study by Hoch et al. [58], protein functionalization was performed with a ratio of 1:2 of gelatin amino groups to methacrylation agents; their study showed that methacrylation primarily occurred via amine groups. However, when they performed methacrylation with a ratio of 1:10, they observed a sharp peak at 6.1 ppm, suggesting that methacrylation might also occur through other functional groups, such as hydroxyl groups. Furthermore, at a 1:2 ratio, the degree of methacrylation was calculated to be around 80% using the TNBS method and approximately 85% using the ^1^H-NMR method. Excessive use of methacrylation agents (such as 1:10) resulted in an almost 100% conversion of free amino groups.

### 3.2. Fourier Transform Infrared Spectroscopy (FTIR)

Figure 2c displays the FTIR spectra of the GelMA, KerMA, GelMA-KerMA, GEN@PVA/GelMA-KerMA, FGF-2@PVA/GelMA-KerMA and FGF-2@GEN@PVA/GelMA-KerMA patches. The FTIR spectrum of GelMA demonstrates peaks at 1629 cm^−1^ for the C=O stretching groups in the Amide I band, at 1535 cm^−1^ for the N–H bending groups in the Amide II band, and at 1241 cm^−1^ for the C–N stretching and N–H bending in the Amide III band [54,59]. The peak noted in the range of 3200–3400 cm^−1^ corresponds to peptide bonds (N–H stretching) as well as -OH functional groups [59,60]. For the KerMA spectrum, the absorption band at 1625 cm^−1^ is assigned to the C=O stretch (Amide I), the peak at 1527 cm^−1^ to N–H bending (Amide II), and the peak at 1240 cm^−1^ to C–N stretching and N–H bending (Amide III) [59]. The peak detected at 3272 cm^−1^ is attributed to stretching vibrations of O–H and N–H (Amide A); the peak at 2925 cm^−1^ is associated with the symmetrical CH_3_ stretching vibration [61]. Amide I–III bands offer crucial insights into the protein conformation and changes in the backbone structure of proteins. According to the information in the literature, peaks at around 3270 cm^−1^ (Amide A) are associated with a α-helix structure, peaks between 1539 and 1515 cm^−1^ (Amide II) to a β-sheet structure, and peaks at about 1625 cm^−1^ (Amid I) to a α-helix and β-sheet combination [62,63]. Also, the peak around 1240 cm^−1^ (Amide III) can be attributed to the β-sheet structure [63]. For the GelMA-KerMA spectrum, the presence of the characteristic FTIR bands of GelMA and KerMA confirms the successful incorporation of KerMA into GelMA [64]. When the spectra of GEN@PVA/GelMA-KerMA, FGF-2@PVA/GelMA-KerMA, and FGF-2@GEN@PVA/GelMA-KerMA were examined, the coating of GelMA-KerMA surface with GEN@PVA and FGF-2@PVA nanoparticles caused slight shifts in the peaks. However, characteristic peaks of GEN and FGF-2 could not be detected. This result can be attributed to the low presence of GEN@PVA and FGF-2@PVA nanoparticles on the surface of the GelMA-KerMA patches.

### 3.3. Morphological Analysis of GelMA-KerMA Patches

**Figure 3:**
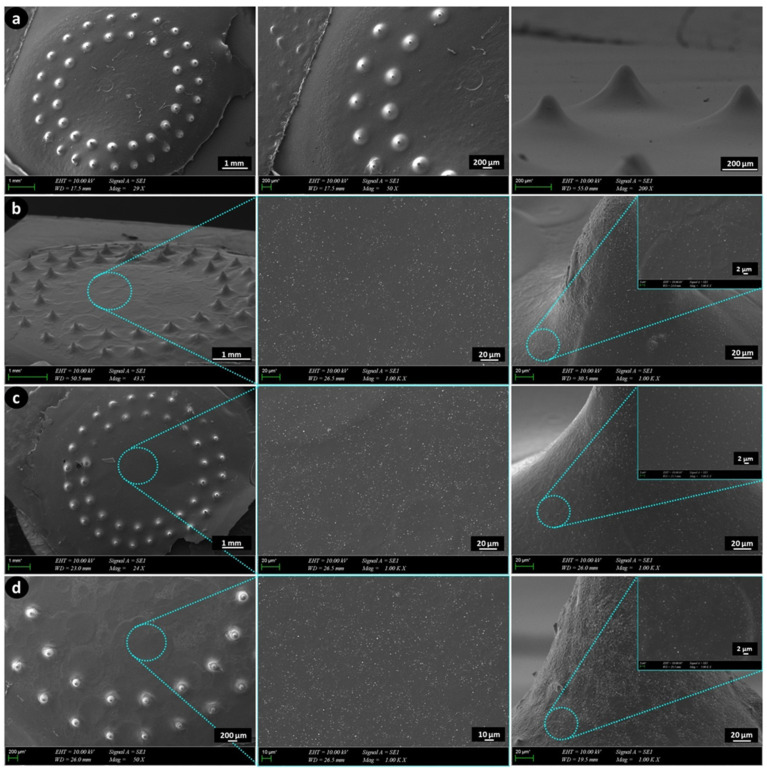
SEM images of 3D-printed GelMA-KerMA patches: (**a**) blank GelMA-KerMA patch, (**b**) GEN@PVA/GelMA-KerMA patch, (**c**) FGF-2@PVA/GelMA-KerMA, (**d**) FGF-2@GEN@PVA/GelMA-KerMA patch.

GelMA-KerMA composite patches equipped with microneedles were produced using a DLP-based 3D printing method following exposure to 90 s of UV light. The obtained 3D-printed GelMA-KerMA patches were coated with GEN@PVA and FGF-2@PVA nanoparticles singly and coaxially for 3 h. Figure 3 presents the SEM images of the GelMA-KerMA, GEN@PVA/GelMA-KerMA, FGF-2@PVA/GelMA-KerMA, and FGF-2@GEN@PVA/GelMA-KerMA patches, respectively. All GelMA-KerMA patches were 10 mm in diameter and 100 µm in height; they contained microneedles in a conical form of approximately 200 µm in height. SEM analysis confirmed that the microneedle-equipped patches have a uniform and regular morphology and do not contain air bubbles or agglomerations. Although there is widespread agreement that the distribution of thickness values in different locations of the TM is not homogeneous, a value varying from 30 to 150 µm for the thickness of the human TM is generally adopted [65,66,67]. The GelMA-KerMA patches possess an appropriate thickness; the microneedles possess an appropriate height for the patch to enter the human TM. The patches depicted in Figure 3b–d showed a more textured surface morphology than the blank GelMA-KerMA patch (Figure 3a), suggesting the existence of nanoparticles. SEM images of Figure 3b–d at high magnifications suggest that the nanoparticles are homogeneously distributed over the patches and have a spherical morphology without fiber formation. The average particle diameter of the nanoparticles on the patches was measured as 218 ± 0.94 nm for GEN@PVA (Figure 3b), 233 ± 0.86 nm for FGF-2@PVA (Figure 3c), and 242 ± 0.11 nm for GEN@FGF-2@PVA (Figure 3d). The possible reason why GEN@PVA nanoparticles are smaller in average particle diameter than FGF-2@PVA may be because the electrostatic interaction between PVA and GEN is stronger than that with FGF-2 [68]. Measurements obtained as a result of coaxial electrospraying exhibited a higher average particle size, confirming the core–shell structure compared to single axial spraying. As a result, these findings are acceptable and widely regarded for the utilization of nanoparticles in drug delivery systems [69,70].

### 3.4. Mechanical Characteristics of GelMA-KerMA Hydrogels

**Figure 4:**
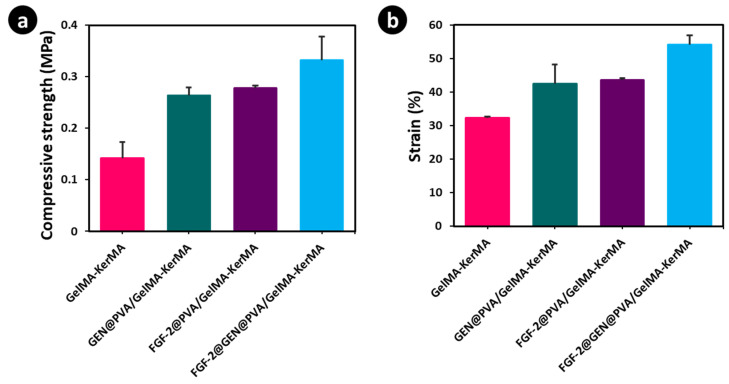
Mechanical properties of blank and nanoparticle-coated 3D-printed GelMA-KerMA hydrogels: (**a**) compressive strength, (**b**) strain (%). Data are expressed as mean ± standard deviation (SD, *n* = 3).

The mechanical characteristics of scaffolds significantly affect the morphology, motility, and differentiation of cells [71]. Biomaterials that closely mimic the mechanical properties of the extracellular matrix are highly beneficial in the field of tissue engineering [72]. The mechanical properties of the obtained blank and nanoparticle-coated GelMA-KerMA hydrogels are shown in Figure 4. As noted in Figure 4a, the compressive strengths of the FGF-2@GEN@PVA/GelMA-KerMA (0.33 ± 0.04 MPa), FGF-2@PVA/GelMA-KerMA (0.27 ± 0.004 MPa), and GEN@PVA/GelMA-KerMA (0.26 ± 0.01 MPa) hydrogels were higher than that of blank GelMA-KerMA (0.14 ± 0.03 MPa). Also, similar trends were detected for strain (%) values of GelMA-KerMA hydrogels (from 32.3 ± 0.27 to 54.07 ± 2.7%, Figure 4b). Figure 4a,b demonstrates that the compressive strength and strain (%) values were noticeably high for nanoparticle-coated GelMA-KerMA hydrogels in comparison to blank GelMA-KerMA. The compressive moduli of the blank GelMA-KerMA, GEN@PVA/GelMA-KerMA, FGF-2@PVA/GelMA-KerMA, and FGF-2@GEN@PVA/GelMA-KerMA hydrogels were calculated as 0.09 ± 0.01 MPa, 0.16 ± 0.03 MPa, 0.17 ± 0.04 Mpa, and 0.2 ± 0.04 MPa, respectively. This result confirms that nanoparticles can enhance the mechanical strength of hydrogels through physical interactions [73,74]. Based on these findings, it can be suggested that the nanoparticle coating can improve the mechanical properties and physiological stability of GelMA-KerMA hydrogels in order to adapt to the repair of TM perforations.

### 3.5. Swelling and Degradation Analysis for GelMA-KerMA Patches

**Figure 5:**
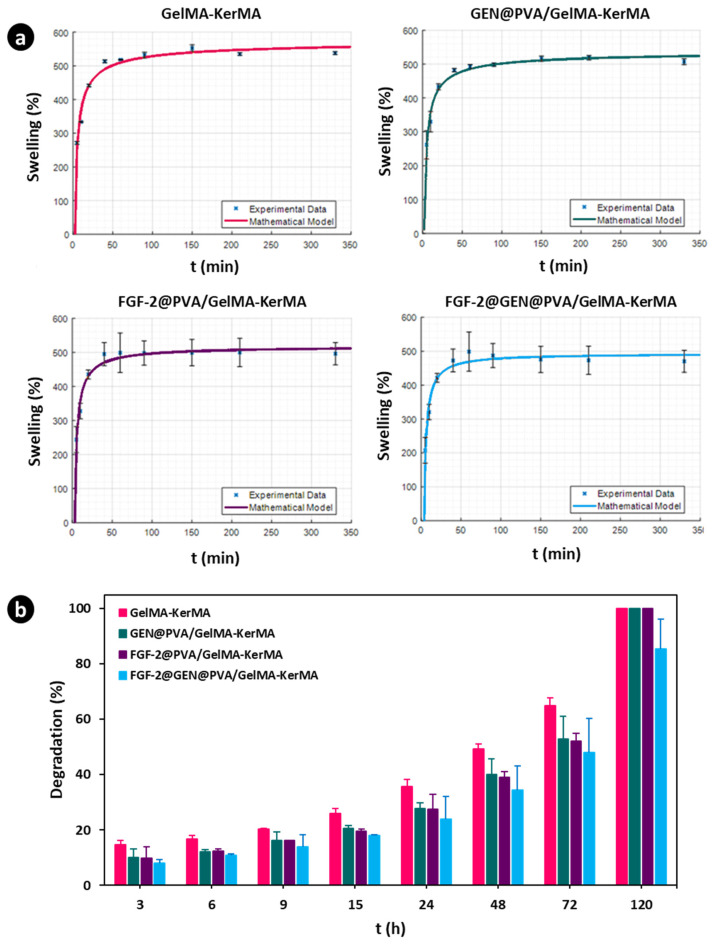
(**a**) Swelling ability of GelMA-KerMA patches incubated in PBS for different time intervals in comparison with the constructed models. (**b**) Degradation profiles of GelMA-KerMA patches. Data are expressed as mean ± standard deviation (SD, *n* = 3).

The swelling ability is a critical factor as it affects the physical and mechanical characteristics of crosslinked composite hydrogels (e.g., diffusion of solutes, surface properties, and surface mobility) [75]. Blank GelMA-KerMA, GEN@PVA/GelMA-KerMA, FGF-2@PVA/GelMA-KerMA, and FGF-2@GEN@PVA/GelMA-KerMA patches were immersed in PBS and placed within an incubator to simulate the in vivo physiological condition. Figure 5a depicts the swelling ability of GelMA-KerMA composite patches. All patches rapidly sucked in PBS and reached an equilibrium phase after 40 min. It was detected that the GelMA-KerMA patches (537.7 ± 4.4%) exhibited a higher swelling ratio compared with GEN@PVA/GelMA-KerMA (507.1 ± 8.8%), FGF-2@PVA/GelMA-KerMA (497 ± 32.5%) and FGF-2@GEN@PVA/GelMA-KerMA (470.4 ± 30.6%). The results indicated that the swelling ratio of blank GelMA-KerMA patches is suppressed by coating their surface with nanoparticles. A possible explanation for this result is that more hydrogen bonds are formed in the nanoparticle-coated patches, which restricts the swelling of the hydrogels [76]. Accordingly, coating the surface of GelMA-KerMA patches with nanoparticles improved the mechanical properties and reduced the water uptake capacity of the resultant hybrid matrices. Furthermore, the kinetics of the swelling were also investigated. The constructed model parameters and model performance data are reported in Table 2. The parameters indicate a Fickian diffusion into the hydrogels as m is greater than 0.5. The *S_e_* values are slightly higher than the experimental equilibrium values since they are the horizontal asymptotes that the model approaches at *t* = ∞. The obtained *S_e_* values also confirm that the water uptake capacity is reduced by the coatings. The performance metrics of all of the constructed models show that all the models are valid within the experimental range of this study. The constructed models are also illustrated in Figure 5a in comparison with the experimental data.

Hydrogels must exhibit suitable degradation behavior to provide sufficient space to allow cell growth and tissue formation for tissue engineering applications [77]. This parameter should be considered for drug delivery applications involving hydrogels, as it can affect their release profiles [78]. The degradation tests were performed in PBS at 37 °C; the degradation profiles of composite patches are displayed in Figure 5b. It was found that swelling reached equilibrium in about 2 h, while the degradation data were collected after 3 h and up to 120 h. As can be seen, blank GelMA-KerMA patches showed stronger degradation behavior compared to the other groups. Blank GelMA-KerMA, GEN@PVA/GelMA-KerMA, and FGF-2@PVA/GelMA-KerMA patches were 100% degraded after 120 h of immersion, whereas FGF-2@GEN@PVA/GelMA-KerMA patches exhibited a slower degradation behavior, and the weight loss reached 85.5 ± 10%. It can be determined from this result that the degradation of GelMA-KerMA patches is controlled by the nanoparticle coating on their surfaces. The results from the swelling and degradation experiments indicated that the extensively swellable matrix interacts with a greater number of water molecules, leading to faster degradation of the system. The hydrolytic cleavage of ester linkages in the hydrogel leads to decreased crosslink density; this phenomenon can be monitored with corresponding increases in the swelling ratio [79]. Conversely, the matrix with slower degradation demonstrated a reduced rate of swelling [74].

### 3.6. In Vitro GEN Release Study

**Figure 6:**
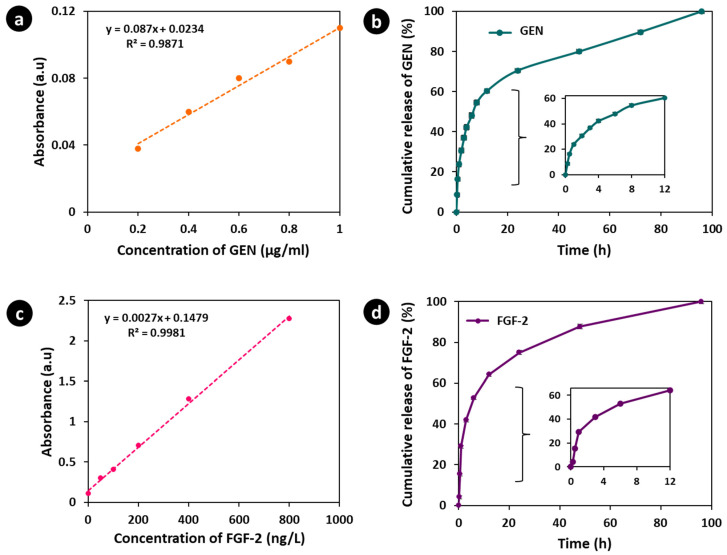
(**a**) Linear calibration curve of GEN. (**b**) The in vitro release profile of GEN from GEN@PVA/GelMA-KerMA patches. The first 12 h GEN release is detailed on the graph. (**c**) Linear calibration curve of FGF-2. (**d**) The in vitro release profile of FGF-2 from FGF-2@PVA/GelMA-KerMA patches. The first 12 h FGF-2 release is detailed on the graph. All the measurements were repeated three times; the errors were less than 5%.

In vitro drug release studies were undertaken to evaluate the release characteristics of GEN from GEN@PVA/GelMA-KerMA patches. The release profiles of GEN@PVA/GelMA-KerMA patches were evaluated under simulated physiological conditions in PBS (pH 7.4) and at 37 °C. UV spectra were recorded within the GEN concentration range of 0.2 to 1 µg/mL; the presence of released GEN was detected at a UV absorbance of 195 nm. A linear standard calibration curve (*R*^2^ = 0.9871) was created from the GEN absorption values (Figure 6a). The cumulative release of GEN from GEN@PVA/GelMA-KerMA patches is presented in Figure 6b. GEN@PVA/GelMA-KerMA patches demonstrated a 60.4 ± 1.3% burst release of GEN from the patches within the first 12 h of incubation. Due to the hydrophilicity of the PVA polymer [80] and the highly water-soluble nature of GEN [81], patches that became more sensitive to environmental factors caused a rapid release of the antibiotic. Following the initial burst release, the drug exhibited a slow and controlled release. At the end of 96 h of incubation, 100% of the GEN was released from the patches.

The rate of drug release is significantly influenced by the physical properties of the drug, including factors such as drug solubility and drug–polymer interactions [82]. PVA is a polymer that is ideal for drug release since the hydroxyl groups in its structure can interact with the functional groups of the drug through secondary interactions such as hydrogen bridges [83]. One of the other factors affecting drug release is the concentration of the polymer. The increase in the concentration of the PVA solution may contribute to the prolongation of drug release as well as the structuring of the polymer [84]. At this point, the drug is expected to have a slower release rate in carriers with a higher concentration of PVA. The duration of drug release can be regulated by varying the nanoparticle coating time as well [85]. For this study, GelMA-KerMA patches were coated with GEN-loaded PVA NPs for 3 h; therefore, the GEN release time from the patches can be increased by increasing the coating time. Another factor affecting drug release is the use of various polymers to improve the mechanical properties of GelMA to meet the needs of a controlled drug release [86]. It was observed that the drug released from GelMA-based composite hydrogels provided a more sustained drug release compared to GelMA hydrogel [87]. The KerMA compound in the patch is predicted to increase the drug release duration in GEN@PVA/GelMA-KerMA patches.

Dizaj et al. [88] found that pure GEN was completely released in 45 min; the release of the drug from CaCO_3_ nanoparticles was prolonged for up to 12 h. In another study, Abd-Elhakeem et al. [89] loaded chitosan nanoparticles with GEN and ascorbic acid and reported that the drugs were rapidly released in the first 10 h and exhibited a slow release until they reached the highest amount after about 60 h. PVP is a hydrophilic polymer that is associated with the rapid dissolution and release of the drug (similar to PVA) [90]. Ali et al. [91] coated microneedles with PVP and PCL particles using the EHDA method. The in vitro drug release studies indicated 100% drug release within 120 min for PVP particles, while 100% drug release occurred after 7 days for PCL particles.

### 3.7. In Vitro FGF-2 Release Study

The in vitro release profile of FGF-2 from FGF-2@PVA/GelMA-KerMA patches was determined using an ELISA kit. A linear standard calibration curve (*R*^2^ = 0.9981) obtained from the FGF-2 absorbance values is shown in Figure 6c. As presented in Figure 6d, a 64.2 ± 0.5% burst release of FGF-2 from the patches was seen within the first 12 h. These results clearly indicate that FGF-2 is susceptible to the ionic strength associated with the environment, leading to rapid release [92]. After 96 h of incubation, the patches completely released (100%) the FGF-2.

Similar to our findings, Mabilleau et al. [93] incorporated FGF-2 into p(HEMA-co-VP) cylinders to enhance bone growth and found that all FGF-2 was released from the hydrogel after 5 days. In another study, Fathi and colleagues [94] used a PVA–dextran blend hydrogel for the controlled delivery of FGF-2. They reported a rapid release of FGF-2 in the initial burst phase, followed by a steady and leisurely release over longer periods (up to 400 h). The initial burst effect was attributed to the closer localization of FGF-2 to the hydrogel surface during the loading process [94]. In a recent study, Luo et al. [95] loaded FGF-2 into GelMA hydrogels at different concentrations and observed a burst release of FGF-2 from the hydrogels within the first 5 days. GelMA hydrogels were capable of forming polyionic complexes with growth factors, resulting in sustained growth factor release. As the GelMA macromer concentration increased in the hydrogels, the release rate of FGF-2 decreased [95].

### 3.8. GEN Release Kinetics

The release kinetics of GEN from GEN@PVA/GelMA-KerMA patches were analyzed using Korsmeyer–Peppas, zero-order, first-order, Higuchi, and Hixson–Crowell release models. Table 3 displays the kinetic constants and regression coefficients (*R*^2^) that were obtained from the graphs for the GEN@PVA/GelMA-KerMA patches. The Korsmeyer–Peppas model demonstrates the best compliance with a higher *R*^2^ value (*R*^2^ = 0.9758) for the GEN@PVA/GelMA-KerMA patches. Furthermore, “n” values associated with various transport mechanisms were correlated in accordance with the Korsmeyer–Peppas model. The ranges of “n” values describing the mechanism of drug release from polymeric materials are shown in Table 4 [96]. Considering Table 3, the “n” value for the GEN@PVA/GelMA-KerMA patches is greater than 1; this result indicates that the GEN is released from the patch via the Super Case II transport mechanism, possibly because of swelling and chain disentanglement of the hydrophilic polymer [97].

### 3.9. In Vitro Antimicrobial Activity

**Figure 7:**
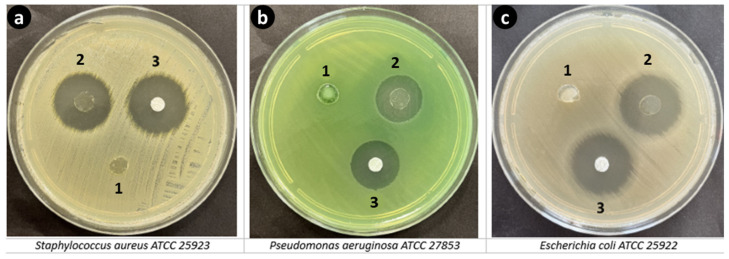
Antibacterial activities of GelMA-KerMA and GEN@PVA/GelMA-KerMA patches against (**a**) *S. aureus*, (**b**) *P. aeruginosa* and (**c**) *E. coli*. (1) Blank GelMA-KerMA, (2) GEN@PVA/GelMA-KerMA, and (3) GEN (positive control).

The antibacterial functionality of GelMA-KerMA and GEN@PVA/GelMA-KerMA patches was assessed by a disc diffusion test. The disc diffusion test revealed that GEN@PVA/GelMA-KerMA patches displayed antibacterial properties against *S. aureus*, *P. aeruginosa*, and *E. coli*. In contrast, as expected, the blank GelMA-KerMA patch showed no antibacterial activity (Figure 7). The inhibition zone diameters detected from GEN@PVA/GelMA-KerMA patches are detailed in Table 5.

### 3.10. Cell Culture Studies

**Figure 8:**
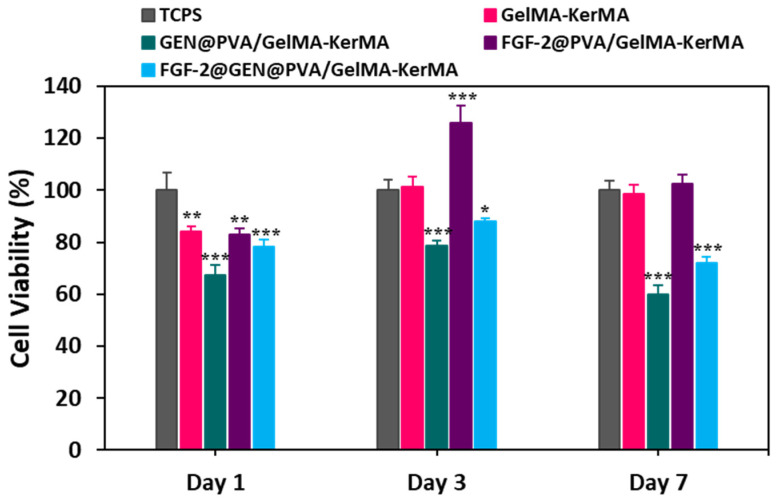
Cell viability results of 1-day, 3-day, and 7-day treated GelMA-KerMA patches. The TCPS was accepted as the control. (* *p* ≤ 0.05, ** *p* < 0.01, *** *p* < 0.001; data presented are mean ± SD, *n* = 3).

GelMA hydrogels and their combined derivatives are widely preferred in tissue engineering due to their attractive properties, such as good biocompatibility, strong cell adhesion, and tunable physicochemical properties [98]. In this study, the biocompatibility of GelMA-KerMA, GEN@PVA/GelMA-KerMA, FGF-2@PVA/GelMA-KerMA, and FGF-2@GEN@PVA/GelMA-KerMA patches were evaluated by MTT assay using hADMSCs after 1, 3 and 7 days of incubation (Figure 8). The percentages of cell viability on blank GelMA-KerMA patches were 84.2 ± 2.01%, 101.1 ± 4.09%, and 98.5 ± 3.53% for 1, 3 and 7 days, respectively. In the GEN@PVA/GelMA-KerMA patch group, cell viability increased on the third day of culture (78.8 ± 1.65%) and decreased slightly on the seventh day (60 ± 3.22%). This reduction in the rate of cell proliferation from day 1 to day 7 could be due to the negative effect of GEN on cell growth and differentiation [99]. The negative effect of the drugs on cell viability and adhesion is linked to the effect of the antibiotic agents on the mitochondrial activities of cells [100]. The FGF-2 was noted to regulate cellular functions such as cell migration, cell proliferation, and cell differentiation. The FGF-2@PVA/GelMA-KerMA patch group had a positive effect on cell viability, with values of 125.8 ± 6.83% at day 3 and 102.51 ± 3.67% at day 7. Compared to the control group, this group significantly promoted cell growth because of the proliferative properties of FGF-2 [101]. The cell viability in the FGF-2@GEN@PVA/GelMA-KerMA patches was measured as 78.2 ± 2.75%, 87.92 ± 1.44% and 72.12 ± 2.37% on days 1, 3 and 7, respectively. This slight decrease in cell viability could also be due to the negative effect of GEN, as mentioned before.

**Figure 9:**
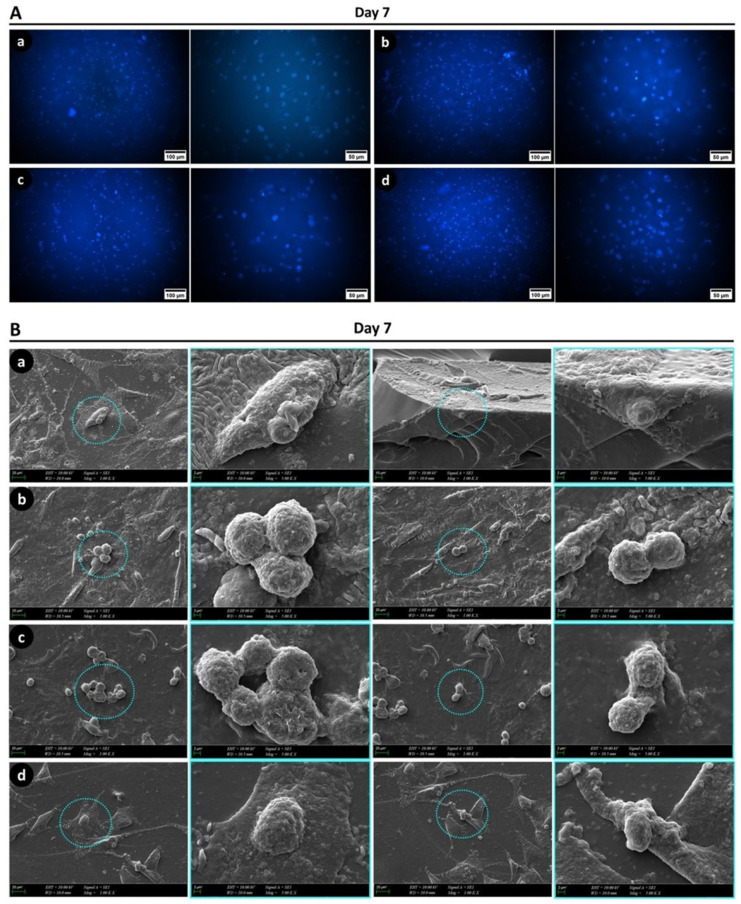
(**A**) Fluorescence images of the GelMA-KerMA patches on the 7th day of cell growth: (**a**) blank GelMA-KerMA patch, (**b**) GEN@PVA/GelMA-KerMA patch, (**c**) FGF-2@PVA/GelMA-KerMA, (**d**) FGF-2@GEN@PVA/GelMA-KerMA patch. (**B**) SEM images of the cultured GelMA-KerMA patches on the 7th day of cell growth: (**a**) blank GelMA-KerMA patch, (**b**) GEN@PVA/GelMA-KerMA patch, (**c**) FGF-2@PVA/GelMA-KerMA, (**d**) FGF-2@GEN@PVA/GelMA-KerMA patch. Blue circles indicate round-shaped cells in all groups.

Fluorescence images of DAPI-stained hADMSCs on GelMA-KerMA patches after 7 days of incubation are provided in Figure 9A. As observed in the figure, the cells were able to attach and proliferate on each patch for the one-week culture period. The images revealed no notable differences in cell density between patches.

The morphology of hADMSCs on the patches was examined with SEM after 7 days of incubation. As shown in Figure 9B, the cells exhibited robust adhesion and migration on the surfaces of all GelMA-KerMA patches. Additionally, both round and spindle-like shapes were observed in the cells of all groups, suggesting the cells were actively growing on the patches. Specifically, the cells on the FGF-2@GEN@PVA/GelMA-KerMA patch displayed a well-spread morphology with numerous filopodia, and cell–cell interactions were more evident compared to other groups. This could be attributed to the enhanced cell adhesion and growth favored by the uneven surface created by the particle coating [102]. Based on SEM observations, it is clear that all patches provided favorable environments for the growth, proliferation, and migration of cells. Consequently, in vitro cell culture studies showed that all GelMA-KerMA patches were biocompatible with hADMSCs, and they promoted cell attachment and proliferation without any cytotoxic effect over the 7-day incubation period.

## 4. Conclusions

This study presents the design and fabrication of GelMA-KerMA composite patches for the potential treatment of TM perforations using tissue engineering principles. The GelMA-KerMA patches, developed by the DLP 3D printing method, were biofunctionalized by coating them with nanoparticles containing GEN and FGF-2. ^1^H NMR analysis verified the presence of methacryloyl groups for both GelMA and KerMA. SEM analysis proved that the 3D-printed GelMA-KerMA patches with microneedles were effectively fabricated in the appropriate size and geometry and that the nanoparticles with spherical morphology were homogeneously dispersed on the patches. Furthermore, mechanical tests have shown that the mechanical properties of the patches were enhanced as a result of the nanoparticle coating. Additionally, it was observed that the water uptake capacity of GelMA-KerMA patches decreased, and their degradation slowed down with nanoparticle coating. According to in vitro release studies, both GEN@PVA/GelMA-GelMA-KerMA and FGF-2@PVA/GelMA-KerMA patches showed burst release within the first 12 h (GEN: 60.4 ± 1.3%, FGF-2: 64.2 ± 0.5%). The in vitro antimicrobial tests confirmed that GEN@PVA/GelMA-KerMA patches exhibited significant antibacterial activity against *P. aeruginosa*, *S. aureus*, and *E. coli*. As proven by the cell proliferation analysis, none of the patches had cytotoxic effects on hADMSCs, which was confirmed by fluorescence microscopy and SEM analysis, showing the patches supported cellular attachment and proliferation over 7 days of incubation. Overall, the findings of our study suggest that nanoparticle-coated 3D-printed GelMA-KerMA patches could serve as an effective approach for the treatment of TM perforations.

## Figures and Tables

**Table 1:** Details of the samples used for the electrospray process.

Samples	Process	Shell	Core
GelMA-KerMA	-	-	-
GEN@PVA/GelMA-KerMA	Single	1% (*w*/*v*) PVA 0.2% (*w*/*v*) GEN	-
FGF-2@PVA/GelMA-KerMA	Single	1% (*w*/*v*) PVA FGF-2 (100 ng/mL)	-
FGF-2@GEN@PVA/GelMA-KerMA	Coaxial	1% (*w*/*v*) PVA 0.2% (*w*/*v*) GEN	1% (*w*/*v*) PVA FGF-2 (100 ng/mL)

**Table 2:** Model parameters and performance metrics of the obtained swelling models.

Hydrogels	Model Parameters			Performance Metrics	
	S_e_ (%)	k_s_ (min^m^)	m	*R* ^2^	RMSE
GelMA-KerMA	579.79	872.65	0.6167	0.9668	21.39
GEN@PVA/GelMA-KerMA	541.46	843.30	0.6625	0.9780	26.76
FGF-2@PVA/GelMA-KerMA	519.20	1117.2	0.8453	0.9673	19.69
FGF-2@GEN@PVA/GelMA-KerMA	493.31	1486.6	1.0061	0.9665	20.71

**Table 3:** Results of mathematical drug release models of GEN@PVA/GelMA-KerMA patches.

	Korsmeyer-Peppas		Zero-Order		First-Order		Higuchi		Hixson-Crowell	
	*R* ^2^	*n*	*R* ^2^	*K*_0_	*R* ^2^	*K*_1_	*R* ^2^	*K_h_*	*R* ^2^	*K_hc_*
GEN@PVA/GelMA-KerMA	0.9758	39.054	0.71	0.5061	0.9531	−0.01	0.943	13.7	0.9351	0.0228

**Table 4:** Transport mechanism types according to the range of the *n* value.

The Ranges of *n* Values	Transport Mechanisms
0.45 ≤ *n*	Fickian diffusion mechanism
0.45 < *n* < 0.89	Non-Fickian transport
*n* = 0.89	Case II (relaxational) transport
*n* > 0.89	Super Case II transport

**Table 5:** Inhibition zone diameters of GelMA-KerMA and GEN@PVA/GelMA-KerMA patches against *S. aureus*, *P. aeruginosa*, and *E. coli*.

	Blank GelMA-KerMA	GEN@PVA/GelMA-KerMA (15 µg)	GEN (10 µg Disc)
*S. aureus* 25923	-	25	25
*P. aeruginosa* 27853	-	20	21
*E. coli* 25922	-	25	26

## Data Availability

The datasets generated during and/or analyzed during the current study are available from the corresponding author on reasonable request.
